# Animal-to-Human Translation Difficulties and Problems With Proposed Coding-in-Noise Deficits in Noise-Induced Synaptopathy and Hidden Hearing Loss

**DOI:** 10.3389/fnins.2022.893542

**Published:** 2022-05-23

**Authors:** Sara Ripley, Li Xia, Zhen Zhang, Steve J. Aiken, Jian Wang

**Affiliations:** ^1^School of Communication Sciences and Disorders, Dalhousie University, Halifax, NS, Canada; ^2^Department of Otolaryngology-Head and Neck Surgery, Mianyang Central Hospital, School of Medicine, University of Electronic Science and Technology of China, Mianyang, China; ^3^Department of Otolaryngology-Head and Neck Surgery, Shanghai Jiao Tong University Affiliated Sixth People’s Hospital, Shanghai, China; ^4^Otolaryngology Institute of Shanghai Jiao Tong University, Shanghai, China

**Keywords:** noise induced synaptopathy (NIS), ribbon synapses, temporal processing, coding-in-noise deficit, cochlear efferent, fluctuation profile, auditory nerve

## Abstract

Noise induced synaptopathy (NIS) and hidden hearing loss (NIHHL) have been hot topic in hearing research since a massive synaptic loss was identified in CBA mice after a brief noise exposure that did not cause permanent threshold shift (PTS) in 2009. Based upon the amount of synaptic loss and the bias of it to synapses with a group of auditory nerve fibers (ANFs) with low spontaneous rate (LSR), coding-in-noise deficit (CIND) has been speculated as the major difficult of hearing in subjects with NIS and NIHHL. This speculation is based upon the idea that the coding of sound at high level against background noise relies mainly on the LSR ANFs. However, the translation from animal data to humans for NIS remains to be justified due to the difference in noise exposure between laboratory animals and human subjects in real life, the lack of morphological data and reliable functional methods to quantify or estimate the loss of the afferent synapses by noise. Moreover, there is no clear, robust data revealing the CIND even in animals with the synaptic loss but no PTS. In humans, both positive and negative reports are available. The difficulty in verifying CINDs has led a re-examination of the hypothesis that CIND is the major deficit associated with NIS and NIHHL, and the theoretical basis of this idea on the role of LSR ANFs. This review summarized the current status of research in NIS and NIHHL, with focus on the translational difficulty from animal data to human clinicals, the technical difficulties in quantifying NIS in humans, and the problems with the SR theory on signal coding. Temporal fluctuation profile model was discussed as a potential alternative for signal coding at high sound level against background noise, in association with the mechanisms of efferent control on the cochlea gain.

## Introduction

Noise induced hearing loss (NIHL) is typically defined and quantified by the permanent threshold shift (PTS) caused by noise exposure (Berger et al., 1978). In recent years, however, this concept has been expanded by the finding in animal studies that noise can cause a significant amount of damage to the ribbon synapses between inner hair cells (IHC) and spiral ganglion neurons (SGN) in the cochlea without PTS (Kujawa and Liberman, 2009; Moser et al., 2013; Starr and Rance, 2015; Moser and Starr, 2016; Song et al., 2016; Kaur et al., 2019; Kim et al., 2019; Liu et al., 2019). After a brief, 2-h exposure of noise at 100–106 dB SPL, these studies have reported an initial loss of up to 50% of ribbon synapses. Auditory nerve malfunctions are expected in association with such massive damage and synapse loss, but these could not be detected by routine audiology assessment focused on thresholds because of the absent PTS. Damage and loss of ribbon synapses, as well as associated functional deficits, can be collectively described as noise-induced synaptopathy (NIS) (Chen et al., 2019a). However, before the functional deficits were detailed and the nature of the deficits was uncovered, the concept of noise induced hidden hearing loss (NIHHL) was proposed to umbrella any potential problems resulting from this pathology (Plack et al., 2014; Le Prell and Clavier, 2017; Liberman, 2017; Liberman and Kujawa, 2017; Chen et al., 2019a; Huet et al., 2019; Kohrman et al., 2020). One of the primary potential problems of interest is coding-in-noise deficit (CIND), which describes an impaired ability to perceive sound in background noise. CIND has been speculated as the major problem in subjects with NIHHL or NIS without PTS due to the selective damage and loss of the ribbon synapses innervating auditory nerve fibers with low spontaneous rates (LSR ANFs) by noise exposure and the unique role of LSR ANFs in signal coding against high level background noise.

Noise induced hidden hearing loss has been of special interest in the field of audiology since the first report on the noise induced synaptic loss without PTS in CBA mice (Kujawa and Liberman, 2009), and it continues to gain attraction as noise-induced synaptic damage may also occur in humans.

### Clarification and Differentiation Between Concepts of Noise Induced Synaptopathy and Noise Induced Hidden Hearing Loss

*Some concepts have been used in this field widely, but their definitions may not always be clear and are sometimes misused. For example, the terms NIS and NIHHL are sometimes used interchangeably. It is beneficial to make a clear differentiation between the two. In this review, NIS covers not only the noise induced loss of but also damage to cochlear ribbon synapses, as well as the associated consequences to cochlear function. Moreover, NIS can occur with or without NIHL, which is typically defined by PTS. However, NIS usually refers to cases without PTS in this review, unless otherwise stated. In any case, NIS mainly refers to cochlear pathology. In contrast, NIHHL refers to any hearing problems caused by noise other than hearing loss defined as PTS. It is notable that while NIHHL caused directly by NIS is likely to result from cochlear dysfunction, it could also reflect changes in central mechanisms.*

To date, although there have been a significant number of studies on the topics of NIS and NIHHL, many knowledge gaps remain. The pathology associated with noise-induced synaptic damage and loss is largely understood based on studies using laboratory animals. Since morphological evaluation of cochlear synapses is almost impossible in humans due to ethical limitations, animal data have been used to interpret or predict synaptopathy in humans—a practice that is necessary but not ideal. In doing so, large differences in noise exposures used with animals in laboratory settings and those experienced by human beings have been generally ignored (see details in section “Noise Induced Synaptopathy Studies in Animal Models and Difficulty in Translation” below).

Perceptual difficulty in background noise, which can be referred to as a coding-in-noise deficit (CIND, the term that will be used in this review), has been thought to be the major problem in NIHHL. The theoretical base underlying this idea is the functional categorization of auditory nerve fibers (ANFs) related to spontaneous rate (SR) and the bias of noise damage to the synapses innervating ANFs with low SR (LSR). However, a selective loss of LSR ANFs has only been reported in two animal studies (Furman et al., 2013; Song et al., 2016) and cannot be confirmed in humans due to technical difficulties in recording single unit ANF function. So far, there are no reliable objective measurements that can precisely verify and quantify NIS in humans (see Section “Measurements Based on Middle Ear Muscle Reflex in NIS Detection” below). In behavioral studies attempting to verify CIND in humans with a history of noise exposure but no PTS, contradictory results have been reported (see Section “Is Coding-in-Noise Deficit Really the Major Problem of Noise Induced Synaptopathy and Noise Induced Hidden Hearing Loss?” below). This may be related to technical errors in some cases, but it suggests a larger problem with the idea that a selective loss of LSR ANFs is the primary pathophysiological mechanism underlying NIS and NIHHL.

In this review, previous studies will be summarized to verify the gaps in knowledge associated with the translation from animal models to humans, to clarify relevant concepts and to address existing confusions. The review will re-examine the theory of SR-based functional categories, and the role of ANFs in different SR groups in the coding of high-level sounds against background noise. It will also address challenges with the traditional view and will discuss a new model and its difficulties. Limitations and controversies in studies of NIS and NIHHL will be discussed in detail to facilitate the planning of future research.

## Noise Induced Synaptopathy Studies in Animal Models and Difficulty in Translation

Across species, ribbon synapses between IHCs and SGNs show similarity in their functions and structures (Nouvian et al., 2006; Moser and Starr, 2016; Wagner and Shin, 2019). Moreover, this synapse appears to be universally sensitive to noise damage in the animal models investigated so far. The sensitivity of the ribbon synapses between IHCs and SGNs to noise damage was first investigated by Pujol et al. (1990, 1993, 1996), Puel et al. (1994, 1998), and Pujol and Puel (1999). Due to methodological limitations at the time, synaptic damage from noise or glutamate agonists were considered temporary and therefore did not elicit much attention in the field of hearing research. However, noise damage to this synapse became a hot topic about a decade later following a report in CBA mice showing a significant synaptic loss after a brief noise exposure that did not cause PTS (Kujawa and Liberman, 2009). Unlike the earlier publications, the new research used immunohistology staining against the pre- and post-synaptic structures, which allowed for the counting of synaptic puncta over the whole IHCs so as to quantify the number of synapses (see review Chen et al., 2019a). In the study using CBA mice (Kujawa and Liberman, 2009), the initial loss of the ribbon synapses was more than 60% in the frequency region above 8 kHz after a 2-h expose to a band of noise at 100 dB SPL. The synaptic loss in this mouse strain was largely irreversible with a recovery of less than 10%, leading to a 50% permanent loss in synapses. Interestingly, it was a dominant opinion for many years that noise-induced synaptic loss was irreversible. Connected to this idea, NIS was conceptually narrowed as noise-induced synaptic loss. Lately, however, evidence has accumulated in favor of the idea that noise-induced synaptic loss is largely or partially reversible. A recovery of synaptic counts has been found in guinea pigs (Liu et al., 2012; Shi et al., 2013; Song et al., 2021), rats (Ruttiger et al., 2013; Singer et al., 2013; Bing et al., 2015), and other strains of mice (Shi et al., 2015; Kaur et al., 2019; Kim et al., 2019). Moreover, functional deficits in ANF units have been found to develop with recovery of the synaptic count (Song et al., 2016). This finding is consistent with the idea of synaptic repair and suggests that the repaired synapses are not healthy. In addition, intrinsic mechanisms involving neurotrophins (Sly et al., 2016; Suzuki et al., 2016) and cochlear efferent regulation (Maison et al., 2013; Boero et al., 2018; Ohata et al., 2021) involved in the maintenance and repair of ribbon synapses have been identified (see review Chen et al., 2019a). It is now more accepted that part of the interrupted ribbon synapses can be repaired or re-established, at least partially. It is also possible that damage and repair may occur across surviving synapses. ***Since the repaired/re-established synapses may not be normal but rather have some functional deficits, the concept of NIS should cover not only the loss of synapses, but also the pathology of survived and repaired synapses.***

One of the challenges in human studies of NIS and NIHHL is the difficulty obtaining morphological evidence for cochlear ribbon synapses. Ideally, animal data on cochlear pathology can be used to predict the effects of noise on human cochleae. However, this approach is hampered by a noticeable limitation of the studies using laboratory animals: the type of noise exposure. In order to create a significant amount of damage/loss of synapses, the noise has usually been presented at the highest level possible that does not cause PTS (around 100 dB SPL in mice, and 105 dB SPL in guinea pigs and rats). Noise exposure at such a level can cause a significant amount of synaptic loss within a short period (e.g., 2 h). Moreover, stationary, continuous noise exposure has usually been used. While the animal data suggests the possibility of NIS in humans, direct translation is not valid because the noise used in the animal studies is unlike what humans experience outside of laboratory settings. The noise frequently experienced by humans that has raised the most concern comes from traffic (Munzel and Sorensen, 2017; Nieuwenhuijsen et al., 2017; Zare Sakhvidi et al., 2018; Munzel et al., 2020), recreational events (Ivory et al., 2014; Fulbright et al., 2017), working in industrial settings (Stucken and Hong, 2014; Lie et al., 2016), and military activity (Pfannenstiel, 2014; Nakashima and Farinaccio, 2015). For the purpose of this review, noise related to military activity will not be considered because of its limited relevance to the general population. Several general features differentiate the noise experienced by humans from that used in previous NIS studies with animals. First, the noise produced by traffic, industrial settings, and recreational events is generally of a much lower sound level than what has been used to cause NIS in animal studies, especially when the use of hearing preservation methods/devices is taken into consideration under current safety standards. Currently, safety regulations ensure that the noise levels rarely exceed 90 dB SPL. Furthermore, the long-term equivalent (Leq) sound level of noise generated by traffic is generally lower than 80 dBA, indicating that even though noise levels of traffic may frequently peak at very high levels, those instances will only last for very short periods of time (Jagniatinskisa et al., 2017; Oiamo et al., 2017). Secondly, the noise experienced by humans in real life is temporally fluctuated in level (Barlow and Castilla-Sanchez, 2012; Masullo et al., 2016), not stationary as what is used in the laboratory studies. Thirdly, the noise experienced by humans is generally intermittent or repeated interruptedly, with damaging doses accumulating across long periods of time.

The resting time between the segments of noise exposure obviously allows for the recovery or repair of potential damage and likely changes the consequence of consecutive noise exposure on the synapse. Therefore, the pathology caused by such noise may be different from what is caused by a brief exposure at high level. Related to the level difference in noise exposure, is the need to validate the “equal energy” hypothesis, which is generally accepted for NIHL (Ward et al., 1981; Gomez Estancona et al., 1983; Lindgren and Axelsson, 1983; Roberto et al., 1985; Fredelius et al., 1987; Borg and Engstrom, 1989). However, this hypothesis may not hold in the development of NIS. In one study, noise of 84 dB SPL was presented continuously to CBA mice for 168 h, resulting a much higher total dose than the brief noise of 2 h at 100 dB SPL (Maison et al., 2013). This noise exposure did not cause a significant loss of ribbon synapses compared to the large amount of synaptic loss in the same strain of mice after the brief noise exposure (Kujawa and Liberman, 2009). All these discrepancies make it invalid to predict NIS in humans using the animal data that is currently available.

## How Can Noise Induced Synaptopathy Be Quantified in Humans With Potential Noise Induced Hidden Hearing Loss?

Great efforts have been expended to quantify potential NIS in human subjects. However, efforts in this regard are largely hindered by the fact that it is almost impossible to observe the synaptic status of cochleae directly in humans due to ethical restrictions. Limited post-mortem cochlear analysis has shown synaptic damage in subjects with noise-exposure history but normal hearing thresholds and OHCs (Zeng and Shannon, 1995; Viana et al., 2015). However, the synaptic loss in such samples cannot be fully attributed to noise due to the involvement of aging.

Can the loss of ANFs by synaptic damage be verified functionally? Theoretically, there are many measurements that can quantify the loss of ANF function. However, to do such measurements in a clinically applicable, non-invasive manner appears to be very challenging. Presently, several objective methods have been proposed for detecting NIS in human subjects. Many of them aim to measure the change of transient cochlear responses, while other studies aim to evaluate ANF responses phase-locked to amplitude modulation.

### Measurements Based Upon Transient Responses

Auditory nerve fibers will not function at all when synapses with IHCs are lost and ANFs connected by damaged synapses will have a reduced firing rate in response to sound (Song et al., 2016). Therefore, NIS in NIHHL will reduce cochlear neural output to the auditory brain. For example, a reduction of wave I amplitude of the auditory brainstem response (ABR) has been seen in animal studies of NIS (e.g., Kujawa and Liberman, 2009). In humans, such a reduction has been reported in subjects with tinnitus (Schaette and McAlpine, 2011). However, several issues suggest caution in interpreting this result as a validation of ABR for NIS quantification. Firstly, it is not clear if and how the tinnitus in this study was related to noise exposure and therefore NIS, although noise exposure is one of the major causes of tinnitus. Secondly, wave I may not be ideal for estimating NIS clinically due to its small amplitude (<0.2 μV) and large amplitude variation. These features suggest that the ABR wave I may not be a reliable measurement for identifying NIS.

Several alternative ABR measures have been proposed for NIS detection. Instead of measuring wave I directly, one study reported using the amplitude ratio between the waves V and I (Mehraei et al., 2016). The idea underlying this measurement is that, while wave I is reduced by NIS, wave V is likely not reduced or even increased as the result of increased central gain in subjects with hidden hearing loss (HHL) (Plack et al., 2014). Other alternatives are to measure shifts in wave V latency with masking (Mehraei et al., 2016; Gottschalk and Domschke, 2017) and to measure changes in the ratio between the summating potential (SP) and the compound action potential (CAP) in electrocochleography (ECochG) (Phillipson, 2017; Kara et al., 2020).

Auditory brainstem response- and CAP-based amplitude measurements tend to have poor reliability in humans due to poor signal-to-noise ratios in far field recordings. Currently, clinical ECochG measurement is usually conducted with electrode placed in the external ear canal. While the CAP amplitude obtained in such ECochG is larger than the ABR wave I recorded from scalp electrodes, it is still not adequate for a reliable quantification of NIS in NIHHL. Larger ECochG can be obtained by using an electrode on the tympanic membrane or needle electrodes placed on the cochlear promontory. However, such electrodes are less likely to be accepted by subjects.

It is worth noting that all of these measures focus on transient responses of ANFs to acoustic onsets. This conflicts with the idea that noise exposure primarily damages synapses to LSR ANFs because LSR ANFs do not contribute to the on-responses of ANFs (Bourien et al., 2014). If noise-induced synaptic loss is really limited or biased to LSR ANFs, transient responses should be relatively insensitive.

Even with these limitations, positive results have been reported using ABR to identify reduced cochlear output. For example, reduced wave I amplitude and increased V/I ratio has been found in subjects with a high risk of NIS (Suresh and Krishnan, 2020), replicating similar results in subjects with tinnitus (Schaette and McAlpine, 2011). Additionally, lower ABR wave I amplitudes have been reported in veterans with significant history of noise exposure (Bramhall et al., 2021). In another study, however, the CAP amplitude was not found to be correlated with hearing in noise function (Parker, 2020), although this study did not address NIS explicitly.

### Measurements of Phase Locking for Noise Induced Synaptopathy Evaluation

The second approach to evaluating changes in cochlear function with NIS is to measure phase-locked responses to amplitude modulation, also named envelope following responses (EFR) (Bharadwaj et al., 2015; Shaheen et al., 2015; Galvez-Contreras et al., 2017; Kalia et al., 2017; Kobel et al., 2017; Le Prell and Clavier, 2017). This approach is likely to be superior to measurements based upon transient responses for several reasons: (1) EFR reflects ongoing responses and is not related to the onset of stimulation. (2) Depending on the carrier frequency, different regions of the cochlea can be targeted to ensure testing of ANF function from regions of interest. (3) Unlike transient responses that are generated from all categories of ANFs when tested at high sound levels, EFR can selectively target LSR ANFs by using high-level carriers to saturate HSR ANFs. In this approach, shallow modulation depths are favored (Bharadwaj et al., 2014, 2015; Chen et al., 2019b; Fan et al., 2020), because when AM is presented at high levels, temporal amplitude fluctuations with shallow modulation depth are in the range where HSR ANFs are saturated. (4) The EFR test can be easily combined with masking methods to identify CIND.

Since NIS is thought to disproportionately occur in synapses with LSR ANFs, AM responses tested at high sound levels should be significantly attenuated. LSR fibers are also thought to be more important for signal encoding in high-level background noise (Joris and Yin, 1992; Moser and Starr, 2016; Plack et al., 2016; Kobel et al., 2017; Liberman and Kujawa, 2017), because they are robust with respect to masking (Costalupes, 1985; Young and Barta, 1986). Therefore, AM responses should be better suited to detect coding deficits in noise than transient responses such as ABR and CAP, which are dominated by onset responses from high-SR fibers (Bourien et al., 2014). This inference is supported by a study that found a more robust decrease in EFR phase-locking than in ABR wave I amplitude in CBA mice with cochlear synaptopathy [established by an octave-band noise (8–16 kHz) exposure at 98–99 dB SPL for 2 h; Shaheen et al., 2015].

In this study, Shaheen reported changes in the temporal modulation transfer function (TMTF) in mice with NIS (Shaheen et al., 2015). An AM signal with a high carrier frequency was used to target the cochlear region most likely to have NIS. The EFR was recorded in the far field using scalp electrodes. TMTFs from the control mice showed a bandpass pattern with the best modulation frequency located close to 1 kHz. The ANF origin of this peak was identified by the loss of this peak in the mice exposed to a noise that caused a significant amount of synaptic loss in the high frequency region. Similar changes in TMTF were reported in mice treated with cochlear application of ouabain (Parthasarathy and Kujawa, 2018). In this report, ouabain was applied with a dose that selectively killed LSR ANFs. In one of our previous studies in guinea pigs, we measured EFR in both the near field (recording from a round window electrode) and far field (from a scalp electrode) (Chen et al., 2019b). When a high frequency carrier (16 kHz) was used, a significant reduction in near-field EFR amplitude was seen across a wide range of modulation frequencies (from ∼100 to ∼1000 Hz), suggesting that the damage to ribbon synapses reduced phase locked responses of ANFs in a way that was not selective to modulation frequency. However, such a reduction was not seen in the far-field EFR recorded from the scalp. This result indicates that the sensitivity of the far-field EFR is low.

In human studies, positive reports are available showing a reduction of EFR amplitude in subjects with potential NIHHL (Bramhall et al., 2021). A recent study also proposed recording EFR with multi-band complex tones to measure the impact of NIS on cochlear responses (Wang et al., 2019). This approach likely reduces EFR testing time and allows for the evaluation of NIS across a larger frequency range more efficiently. However, the application of EFR for the purpose of identifying NIS needs to be optimized. A promising new approach involves the measurement of EFR to stimuli with rectangular envelopes, since these should be more sensitive to neural damage and less sensitive to changes in the cochlear amplifier. Several studies suggest that these may be more sensitive to NIS (Verhulst et al., 2018; Vasilkov et al., 2021) and more predictive of CIND (Mepani et al., 2021).

### Measurements Based on Middle Ear Muscle Reflex in Noise Induced Synaptopathy Detection

The middle ear muscle reflex (MEMR) plays a role in protecting the cochlea from damage by loud sounds. This reflex is defined by an increased stiffness of the middle ear ossicular chain due to the contraction of middle ear muscles. In humans, the stapedius muscle is the major player in the MEMR. When it is evoked, excitation of IHCs and SGNs is reduced (Simmons, 1960; Borg, 1968). Since the activation of this reflex depends on the strength of the input from auditory nerves, the loss of ANFs due to synaptopathy may reduce the MEMR. Utilizing the MEMR to detect NIS has recently been explored, and it is a compelling idea considering the measurement of this acoustic reflex can easily and non-invasively be integrated into clinical audiology assessments (Valero et al., 2018).

As outlined by Bharadwaj et al. (2019), the hypothesis that the MEMR can be used as an objective measure of NIS detection stems from the likelihood that LSR ANFs play an important role in the MEMR circuit (Liberman and Kiang, 1984; Rouiller et al., 1986; Kobler et al., 1992; Bharadwaj et al., 2019). Since noise is thought to selectively damage synapses with LSR ANFs (Furman et al., 2013; Song et al., 2016), the MEMR should be weakened in subjects with NIS.

The connection between the loss of ANFs and MEMR function has been examined in animal studies. In one study, reflex growth function was measured in mice with varying degrees of NIS (Valero et al., 2018). To avoid attenuation by anesthesia, the mice were tranquilized briefly with isoflurane to allow for the fixation of plastic couplers in their ear canals with cyanoacrylate (Valero et al., 2016). Their surgically affixed head-plates were then secured atop a freely spinning platform on which they could walk at will; the MEMR was tested 15 min after the isoflurane was removed (Valero et al., 2018). The results indicated that both MEMR threshold elevation and magnitude reduction were scaled linearly with percentage of synapse loss, which ranged from 4 to 50% in the 22–45 kHz region. When the reflex elicitor was filtered to stimulate the region with the most synaptopathy, there was a stronger correlation between MEMR change and synaptic loss. Conversely, the correlation was the weakest in the non-synaptopathic region. Since the MEMR was not eliminated but obtained at higher thresholds even in subjects with 50% loss of ANFs, it is possible that ANFs in all three SR categories drive the MEMR (Valero et al., 2018) and NIS induces MEMR deficits due to the reduced number of ANFs. Therefore, MEMR is likely to be a useful metric of NIS.

Positive results have been found in human studies as well (Wojtczak et al., 2017; Mepani et al., 2020; Shehorn et al., 2020). For example, Shehorn et al. (2020) examined relationships between level-dependent speech intelligibility (rollover) and the wideband MEMR in adult participants aged 21–54 with normal hearing thresholds. The subjects were grouped based upon whether they had sought hearing help. Lifetime noise exposure was determined by self-report *via* the Noise Exposure Structured Interview (NESI), which resulted in marginally higher scores in the help-seeking group. The study found that the MEMR magnitude of help-seeking individuals was weaker. To determine the role of various factors (as covariates), including participant group, gender, age, pure tone average (PTA), tinnitus, ABR wave I amplitude, NESI, the side of the MEMR elicitor and elicitor level on MEMR magnitude (the dependent variable), analyses of covariance (ANCOVAs) using general mixed-effects models were performed. The results showed that the significant predictors were, in order of inclusion, elicitor level, elicitor side, and NESI. There was a significant interaction between NESI and elicitor level: for low-elicitor levels particularly, MEMR magnitude decreased with increasing lifetime noise exposure (Shehorn et al., 2020). This study thus agrees with the abovementioned animal research (Valero et al., 2016, 2018).

On the other hand, other studies have found negative results for the use of the MEMR in NIS. For example, one study showed no evidence in human participants for changes in MEMR threshold or growth related to NESI score when using a contralateral BBN elicitor (Causon et al., 2020). Another study examined the relationship between MEMR thresholds and tinnitus, difficulties with speech perception in noise (SPiN) and noise exposure (Guest et al., 2019a). The results of this work also revealed no relation between MEMR and noise exposure. However, the authors of this study refer to a prior study by Wojtczak et al. (2017), which revealed a large reduction in MEMR amplitude (by a factor of roughly four) in participants with tinnitus compared to a control group. In that study, all participants with tinnitus reported excessive and repeated noise exposure. This highlights the possible impact of methodological differences on the likelihood of detecting a relationship.

### Validation and Comparison Across the Objective Measurements

There are noticeable discrepancies in many of the studies of objective measurements of cochlear synaptopathy. Several studies have been conducted with the intention of examining these discrepancies and offering a comparison of the objective measurements detailed above (Guest et al., 2019a; Kaur et al., 2019; Prendergast et al., 2019).

The work by Guest et al. (2019b) assessed the reliability of seven specific measures that fall within the three types of measurement discussed in sections “Measurements Based Upon Transient Responses,” “Measurements of Phase Locking for Noise Induced Synaptopathy Evaluation,” and “Measurements Based on Middle Ear Muscle Reflex in Noise Induced Synaptopathy Detection.” The measures examined in the study were ABR wave I amplitude, ABR wave I growth, ABR wave V latency shift in noise masking, EFR amplitude, EFR growth with stimulus modulation depth, MEMR threshold and an MEMR across-frequency difference measure. The participants of the study consisted of 30 women aged 18–30 and were of a single sex due to known sex differences in electrophysiological response amplitudes. Each participant attended two test sessions, during which all seven measures were assessed. Pure-tone audiometry and distortion-product otoacoustic emissions (DPOAE) were also assessed during the test sessions, to ensure normal cochlear mechanical function.

In addition to examining the reliability of each measure individually, the study also made 18 comparisons across the proxy measures of synaptopathy. The results of the study indicate that measures of EFR amplitude and MEMR threshold are highly reliable measures in humans. The results also indicate that ABR wave I amplitude can be a highly reliable measure if proper care is taken regarding consistency in electrode placement, participant state, and other factors influenced by the researcher or clinician. It should be noted that clicks were used to elicit the ABR in this study, as well as research-grade recording equipment. If adopting ABR amplitude measures, the authors advised that the investigator assess the reliability of their own ABR measurements due to the lower ABR reliability found in their own work (Guest et al., 2019a). Similar results were found in a study that examined the test-retest reliability of raw measures, which found good reliability in MEMR threshold and moderate reliability in ABR wave I amplitude (Kamerer et al., 2019). However, despite the strong reliability of these raw amplitude and threshold measures, no correlations were observed between any of the proxy measures of cochlear synaptopathy. This broadly suggests that the participants did not possess synaptopathy or that the proxy measures were not sensitive to synaptopathy (Guest et al., 2019b).

In a separate study, proxy measures including ABR and EFR were evaluated by examining the effects of age and noise exposure (Prendergast et al., 2019). This study consisted of 156 participants, all with hearing thresholds within normal limits. Lifetime noise exposure was quantified using a structured interview aimed at determining the amount of time spent in environments with noise exceeding 85 dBA. In addition to ABR and EFR, psychophysical tasks such as interaural phase difference (IPD) and amplitude modulation detection (AMD) thresholds were examined, as well as the co-ordinate response measure (CRM) and digit triplet test (DTT) speech tasks. In short, the results of this study showed no evidence of age- or noise-induced cochlear synaptopathy *via* the proxy measures that were examined. Focusing on EFR and ABR for the purpose of this review, this work found no evidence for a relationship between age or noise exposure and EFR or ABR amplitudes. Therefore, by using these proxy measures, the results suggest that there is minimal effect of recreational noise exposure on auditory function for individuals with normal audiograms, which is inconsistent with the predicted effects of synaptopathy (Prendergast et al., 2019).

At this moment, it is too early to make a clear conclusion regarding which (if any) of the objective measures can be used to reliably verify NIS in humans.

## Is Coding-In-Noise Deficit Really the Major Problem of Noise Induced Synaptopathy and Noise Induced Hidden Hearing Loss?

Coding-in-noise deficit refers to a coding deficit in background noise, specifically when examined with signals presented at relatively high sound levels or speech presented at normal levels with high-level background noise. This deficit has been hypothesized to be the major hearing problem associated with NIS and NIHHL (Furman et al., 2013; Bharadwaj et al., 2014; Plack et al., 2014; Kobel et al., 2017; Le Prell and Clavier, 2017; Liberman, 2017; Liberman and Kujawa, 2017; Hesse and Kastellis, 2019; Huet et al., 2019; De Siati et al., 2020; Hertzano et al., 2020; Henry, 2021), based on the functional categorization of ANFs by SR and the disproportionate impact of noise damage on the synapses innervating LSR ANFs. Compared to ANFs with high SR (HSR), LSR ANFs have higher thresholds and larger dynamic ranges (Liberman, 1978, 1982, 1988; Liberman and Beil, 1979; Taberner and Liberman, 2005), and are therefore more important for signal coding at high sound levels (Schalk and Sachs, 1980; Winter et al., 1990). Moreover, LSR ANFs appear to function better for coding signals masked by high-level background noise (Costalupes et al., 1984). Unfortunately, ribbon synapses innervating this group of ANFs are more sensitive to noise damage (Fucci et al., 1997a,b; Furman et al., 2013; Song et al., 2016).

While the hypothesis sounds reasonable, the supporting evidence is weak. At present, there are no solid data from animal studies showing CIND in subjects with NIS. Our group examined the coding of amplitude modulation in background noise using the envelope-following response recorded from the round window and did not find any differences between the control and the noise-exposed group with significant synaptic loss (Chen et al., 2019b; Fan et al., 2020). A positive result has only been reported in a single study that used a paradigm of pre-inhibition of a startle response to airpuffs. A noise burst presented in a background noise was used as the pre-inhibitor (Lobarinas et al., 2017). The report showed reduced pre-inhibition in the noise-exposed group, suggesting a deficit in hearing the noise burst in the background noise. However, noise-induced damage to ribbon synapses was not documented in the study. It is notable that the coding-in-noise deficit was seen only in the rats that were exposed to the noise at 109 dB SPL, but not at 106 dB SPL, which should have been adequate to produce significant NIS. Two limitations make the data interpretation difficult: (1) the pre-pulse inhibition of the startle responses involves the central auditory system, which may compensate for changes in cochlear function related to synaptopathy; and (2) the signal-to-noise ratio (SNR) between the pre-pulse inhibitor and the masker must be at least 20 dB in order to show clear inhibition in this paradigm. This is much higher than the SNR used in most signal-in-noise tasks, such as speech-in-noise measures conducted at ratios between –10 and +10 dB (Billings et al., 2017; Maamor and Billings, 2017; Best et al., 2018; Billings and Madsen, 2018; Yeend et al., 2018).

In human subjects with histories of noise exposure but normal hearing thresholds, there is a lack of consensus concerning the existence of CIND or hearing-in-noise (HIN) problems as well as a lack of morphological evidence and functional data indicating loss or damage of ribbon synapses. There exist many negative publications (Fulbright et al., 2017; Grinn et al., 2017; Grose et al., 2017; Le Prell and Clavier, 2017; Prendergast et al., 2017a,^2019^; Yeend et al., 2017; Guest et al., 2018, 2019a; Valderrama et al., 2018), while positive reports are also available (Alvord, 1983; Kujala et al., 2004; Stone et al., 2008; Kumar et al., 2012; Stamper and Johnson, 2015; Liberman et al., 2016; Tepe et al., 2017; Meehan et al., 2019). For example, the study by Grinn et al. (2017) looked at the effects of long-term self-reported noise exposure as well as a loud recreational event on several audiologic measurements including ECochG. One of the main findings of the study was that there was no evidence of noise-induced decreases in human CAP amplitude in either the retrospective or prospective analyses. Contrarily, the study by Liberman et al. (2016), which assessed college students categorized into low-risk and high-risk groups based on self-report of noise exposure, found an increased SP/AP ratio in the high-risk group. As shown by the examples above, comparisons across studies are difficult due to differences in methodology and subject characteristics. Inconsistent and unreliable methods for quantifying noise exposure (e.g., self-report measures), coupled with a lack of morphological information renders it impossible to confirm the existence of NIS. Moreover, the methods used to identify CIND vary across different studies, some of which need to be validated (see section “The Role of Temporal Processing Deficits in Coding-in-Noise Deficit” for details). Therefore, it remains a mystery as to whether CIND is the major functional deficit in NIS and NIHHL.

## Questioning the Role of Low Spontaneous-Rate Auditory Nerve Fibers in Coding-In-Noise Deficit

Equivocal results and a lack of consensus from studies investigating CIND in both animals and human studies with (potential) NIS makes it necessary to re-examine the hypothesis that CIND is the major deficit associated with NIS and NIHHL, as well as the theoretical basis of this idea (i.e., that noise damage to ribbon synapses innervating LSR ANFs is the major pathology of NIS without PTS and that LSR ANFs are critical for coding signals at high levels and in background noise).

It is important to note that synaptic damage by noise is biased, but not limited, to synapses innervating low-SR ANFs. Since this group of ANFs constitutes only a small proportion of the total population of ANFs (Liberman, 1978), medium-SR (MSR) and even HSR ANFs are not spared when 50% of synapses are lost following a damaging noise exposure. Secondly, interrupted synapses can be repaired, including those innervating LSR ANFs. In Guinea pigs, a significant reduction of LSR ANFs was observed shortly after a noise exposure that initially destroyed ∼50% of synapses in the high frequency region. However, the percentage distribution of ANFs across SR groups recovered 1 month later in spite of an ∼18% loss in the total number of remaining synapses (Song et al., 2016). This suggests that synapses with low-SR ANFs are also partially re-established.

The idea that the coding of high-level signals relies purely on L/MSR ANFs (because HSR ANFs firing rates are saturated at high levels) has also recently been challenged (Carney, 2018). This idea is based on the assumption that ANFs code sound level *via* average firing rate. This assumption was challenged by many aspects of Carney’s review. For example, for such a coding scheme to work, the increase in the average firing rate with sound level must be larger than the variability change in firing rate with sound level. Since variability also increases with sound level, this would require rate-level functions that accelerate with level to compensate for the variability increase; such rate-level functions are not seen for any type of ANF (Siebert, 1965; Viemeister, 1988; Winter and Palmer, 1991; Delgutte, 1996; Heinz et al., 2001; Colburn et al., 2003; see Carney, 2018 for more details).

Models that combine ANFs of different thresholds and dynamic ranges, and over a range of characteristics frequencies (CFs) have been proposed to explain psychophysical level discrimination, which is roughly constant for wideband sound (Delgutte, 1987; Viemeister and Bacon, 1988; Winter and Palmer, 1991; see review Delgutte, 1996). These models are forced to make problematic assumptions. For example, the wide dynamic range of LSR ANFs does not exist for CFs below 1500 Hz (Winter and Palmer, 1991). The limited dynamic range of low-CF LSR ANFs is consistent with the importance of cochlear compression for creating the wide dynamic range of ANFs at higher CFs (Yates et al., 1992) and with physiological evidence based on ANF responses, suggesting that cochlear gain is relatively low for low CFs (Sewell, 1984; Cooper and Yates, 1994).

## Fluctuation Profile Model for Cochlear Coding of High-Level Sound

**FIGURE 1:**
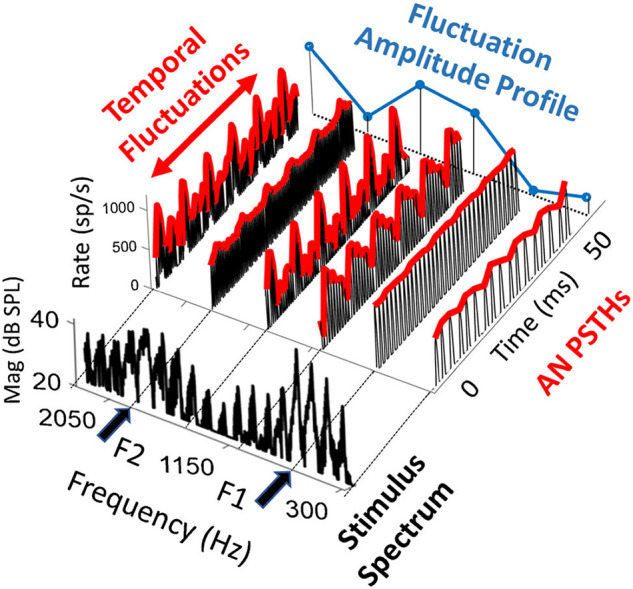
Fluctuation profile of ANF response to vowel /ae/ spectrum, which is in the foreground. The model peristimulus time histograms (PSTHs) of HSR ANFs are presented at formant peaks (F1 = 700 and F2 = 1800 Hz) and troughs. Temporal fluctuation is large at trough frequencies and small nor none at the formants, forming the dips in the fluctuation amplitude that mirrors the formants. Adapted from Carney (2018).

After challenging the idea that the coding of high-level sound relies on LSR ANFs, Carney proposed a model for the coding of high-level spectra *via* HSR ANFs, called the temporal fluctuation profile model (Carney, 2018). Temporal fluctuations exist in complex signals such as speech (Figure 1). For example, in voiced speech sounds, all harmonics are integer multiples of the fundamental frequency (*f*_0_) such that neighboring harmonics are separated by *f*_0_. The amplitude envelope arising from the combination of harmonics is thus modulated at this frequency, giving rise to ANF firing patterns that fluctuate at *f*_0_. For average speech levels (65–70 dB SPL), temporal fluctuation of HSR ANF responses is minimal near spectral peaks (e.g., formants) because they are saturated. However, HSR ANFs in spectral troughs are not saturated and show strong temporal fluctuation by the responses phase-locked to *f*_0_ (e.g., in the single unit study in cats; Schilling et al., 1998). The contrast in fluctuation strength between ANFs tuned to peaks versus trough frequencies gives rise to a temporal fluctuation-profile, which mirrors the spectrum of the voiced speech sound. To make this model work, the signal presentation level must be able to generate differences in temporal fluctuation between spectral peaks and troughs. At very high levels, it is possible that HSR ANF responses will be saturated (and thus non-fluctuating) at both spectral peaks and troughs, such that the model would not work. Therefore, the usefulness of the model appears to be limited to a narrow range of levels (where peaks but not troughs give rise to saturated responses in HSR ANFs).

This model can be used to interpret potential problems in coding speech and other high-level sounds in subjects with NIS. While this model reasonably illustrates the potential contribution of HSR ANFs to the coding of these high-level sounds, the contribution of L/MSR ANFs that are NOT saturated is ignored. When L/MSR ANFs are included, the fluctuation contrast across frequency should be reduced in comparison with a model including only HSR ANFs, because L/MSR ANFs are not saturated and may show little difference in temporal fluctuation between spectral peaks and troughs, at least in healthy cochleae. Interestingly, if NIS is associated with a selective loss or damage to synapses serving L/MSR ANFs, NIS should lead to stronger fluctuation contrast across frequency, thereby predicting better coding for speech. This conflicts with the idea that the damage and loss of L/MSR ANFs in NIS should negatively impact the coding of speech and other high-level sounds; rather, speech coding should be improved by the enhanced fluctuation profile resulting from the lost contribution of L/MSR ANFs.

## The Role of Cochlear Efferent in Noise Induced Synaptopathy and Fluctuation Profile Model

### The Potential Role of Medial Olivocochlear Control in Fluctuation Profile Model

The fluctuation profile appears to be inherited in the midbrain (inferior colliculus, IC), where neurons have response rates that vary systematically with the frequency and amplitude of low-frequency fluctuations on their inputs from cochlear nuclei (CN) (Joris et al., 2004). The low-frequency fluctuations of ANF responses are accentuated by CN neurons which, either directly or *via* other brainstem nuclei, may relay fluctuation profiles to IC neurons, in which the profile is somehow enhanced (Joris et al., 2004). Furthermore, the fluctuation profiles in IC may provide a feedback control mechanism *via* efferent control to the cochlear gain in a way that can possibly enhance the contrast of the fluctuation profile (Carney, 2018).

**FIGURE 2:**
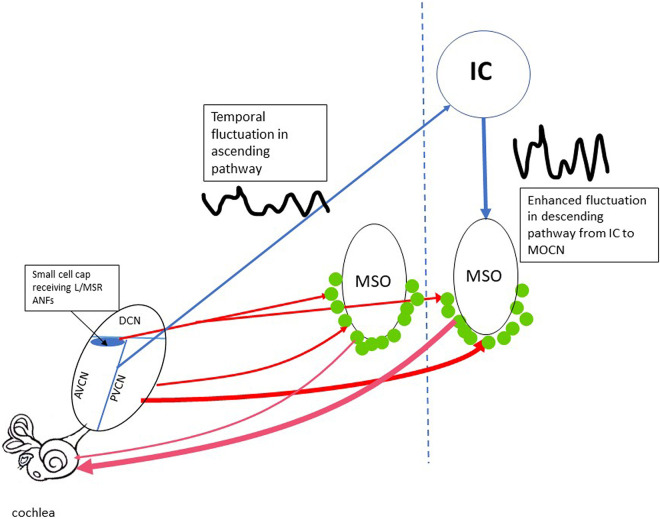
The efferent feedback loops controlling the OHC gain. The short loops going through lower brainstem are marked by redlines. The thickness of the line represents the relative strength in the typical loop from PVCN to MOCNs (green dots). The loop from the small cell cap in AVCN to MOCN is thought to be selective receiving input from L/MSR ANFs. The relative strength of this loop is unknown. The long feedback loop (blue lines) includes the projection from both AVCN and PVCN cores to IC, which is sensitive to the low-frequency temporal fluctuation. The fluctuation is inherited and enhanced in the descending projection from IC to MOCNs. AVCN/PVCN, anterior/posterior ventral cochlear nucleus; DCN, dorsal cochlear nucleus; MOCN, medium olive cochlea neurons; MSO, medium superior olive; IC, inferior colliculus.

Olivocochlear neurons (OCNs) in the lower brainstem are a direct source of cochlear efferent control. They are divided into two groups: medial (MOC) and lateral (LOC) neurons. The function of MOC neurons is better understood; these are known to control OHC gain. Carney’s model proposes two feedback loops for this gain control mechanism as summarized in Figure 2. In the long loop (blue lines in Figure 2), the fluctuation profile established in the ANFs is mapped in CN, which projects to IC and then down to MOC neurons (ANF-CN-IC-MOC-cochlea). In the short loop through the lower brain stem (ANF-CN-MOC-cochlea, red lines in Figure 2), there is a branch receiving projections of L/MSR ANFs in the small cell cap in the marginal shell of the anteroventral cochlear nucleus (AVCN), which then project to MOC neurons (Ye et al., 2000). A large majority of neurons in the small cell cap in cat AVCN have LSRs and very wide dynamic ranges (Ghoshal and Kim, 1996), consistent with the fact that their inputs arise from L/MSR ANFs (Leake and Snyder, 1989; Liberman, 1991; Ryugo, 2008). However, the feedback control in this branch is sensitive to firing rate and not temporal fluctuation. On the other hand, it is widely accepted that the ascending pathway from CN to MOC neurons is mainly through the posterior ventral cochlear nucleus (PVCN) (Figure 2; see review by Guinan, 2006), which is not specific to input from L/MSR ANFs (Thompson and Thompson, 1991; de Venecia et al., 2005; see review Guinan, 2006). In Guinea pigs, lesions in PVCN, but not the other subdivisions, produce long-term decreases in the strength of the MOC-mediated efferent acoustic reflex (EAR). The degree of cell loss within the dorsal part of the PVCN determines the effect of the lesion on the strength of the EAR, as measured in the adaptation of distortion product otoacoustic emissions (DPOAEs) (de Venecia et al., 2005). The authors suggest that multipolar cells within the PVCN have the distribution and response characteristics appropriate to be the MOC reflex interneurons. It is an open question as to whether the PVCN-MOC branch of the lower brainstem loop relies upon input from the LSR ANFs and whether the output of this branch is regulated by efferent projections from fluctuation-sensitive neurons in the IC downstream. There is evidence suggesting that PVCN neurons responsible for the MOC EAR are likely those with chopper histograms and sharp tuning (Brown et al., 2003). However, the SRs and dynamic ranges of these neurons have not been specified.

At this moment, the relative importance between the short and the long loop is unclear. In a study by Gummer et al. (1988) a few medial efferent neurons showed a short latency (5 ms), which is consistent with a direct input from CN neurons to MOC neurons (1988). However, the group delays were longer for most neurons (8.2 ± 1 ms), indicating the involvement of a another relay, likely in the IC (Gummer et al., 1988). Alternatively, the long group delays could be accounted for by a direct CN connection plus a long delay in medial efferent dendrites.

Inferior colliculus neurons are sensitive to low-frequency fluctuations from the ascending pathway (Joris et al., 2004). They have bandpass modulation transfer functions (MTFs) with best modulation frequencies near the fundamental frequency (*f*_0_) of male speech (Krishna and Semple, 2000; Carney et al., 2016); MOC neurons also have bandpass MTFs (Gummer et al., 1988). This indicates that they are likely excited by descending inputs from IC neurons, although it is not clear why the frequencies of human speech would have any relevance for the animals used in those studies. Nevertheless, Carney suggests that the fluctuations in the descending pathway from IC to MOC neurons can enhance the fluctuation profile in ANFs: those ANFs in channels near formant peaks produce less fluctuation, which would result in a weaker MOC excitation through the IC-MOC regulation and then less or no cochlear gain reduction, while those ANFs in channels near troughs produce stronger fluctuations that would excite MOC neurons more strongly, resulting in a greater decrease of cochlear gain. Therefore, the ANFs in the trough channels would be farther away from the level of saturation because of this regulation, while the ANFs in the peak channels would remain saturated.

To make this hypothesis work, one must assume that, when the cochlea is stimulated with temporarily fluctuated signals at a low level, a larger MOC efferent inhibition of cochlear gain would be seen, at least within a certain level range. However, this level effect is opposite to what has been observed in previous studies observing the efferent suppression of otoacoustic emissions and CAP. In such studies, the suppressor is presented contralaterally (contralateral suppression, or CS) for an easy differentiation of the afferent response from the suppressing stimulus. Available data unanimously show larger CS in both OAE (Moulin et al., 1993; Zhang et al., 2007) and CAP (Puria et al., 1996) with a higher suppressor level. However, in those studies, all CS signals are generally stationary. If fluctuation plays a dominant role, as suggested above, the level effect would be opposed by the activity of the long loop when a fluctuated suppressor (such as an AM tone or noise) is used: there would be greater CS for a low-level suppressor (at least within a certain range). However, this may not be seen because efferent control in the short loop through the small cell cap is not determined by temporal fluctuation but rather by overall firing rates of ANF inputs. The efferent control in this pathway should have a larger CS effect at a higher CS level. In one study, efferent suppression of OAEs was observed using an AM signal to evoke contralateral suppression. While the result showed that the suppression was increased with modulation depth, the suppression was observed at only one suppressor level (Maison et al., 1997). Level effects for efferent suppression of OAE and CAP with fluctuated suppressors have never been observed.

Furthermore, speculation about cochlear gain control regulating IHC/ANF saturation conflicts with the fact that the gain reduction is NOT observed for high sound levels but for low levels close to response threshold. This is seen in CAP (Wiederhold and Kiang, 1970) and single ANF responses (Guinan and Stankovic, 1996) evaluated with medial olivocochlear body (MOCB) stimulation, as well as in studies of contralateral suppression of DPOAEs (Chery-Croze et al., 1993; Kujawa et al., 1993; Zhang et al., 2007; Atcherson et al., 2008; Sun, 2008; Chambers et al., 2012; Danesh and Kaf, 2012) and CAP (Kawase and Liberman, 1993; May and McQuone, 1995; Puria et al., 1996; Popelar et al., 2001; Chabert et al., 2002; Najem et al., 2016). Therefore, such gain control is not likely to enhance fluctuation profiles in ANF responses to high-level sound.

### Protective Effect of Efferent on Ribbon Synapses

Evidence is available for the protective role of medial efferent function against noise damage to the synapse. For example, exposure to a noise of 84 dB SPL for 168 h caused a 40% loss of afferent synapses in mice in which surgical de-efferentation to OHCs (not de-efferentation of LOC) was created in the olivocochlear body (OCB) pathway (Maison et al., 2013), while the synaptic loss by this noise was minimal in the control mice. The evidence for MOC protection of OHCs also comes from genetic studies. For example, a point knock-in in a subunit of nicotinic receptor alpha 9 enhanced efferent inhibition and reduced noise induced hearing loss in mice (Taranda et al., 2009; Boero et al., 2018).

While the medial efferent feedback provides EAR *via* the regulation of OHC gain, the functional role of the lateral efferents targeting afferent terminals underneath IHCs is much less understood. However, a few studies have provided positive data indicating a protective role of the lateral efferents. Noise exposure has been found to reduce the strength of OC function (Sliwinska-Kowalska and Kotylo, 2002; Peng et al., 2010). This reduction is likely related to noise-induced damage to the afferent system (such as NIS, which weakens the EAR circuit). Other results have suggested a protective effect against noise damage by LOC fibers. Evidence shows that dopaminergic LOC fibers may exert tonic inhibition to prevent excitotoxicity (Ruel et al., 2001). Moreover, selective removal of LOC neurons has shown to increase cochlear nerve excitotoxicity (Darrow et al., 2007; Lendvai et al., 2011).

## The Role of Temporal Processing Deficits in Coding-In-Noise Deficit

### Temporal Processing Disorders in Noise Induced Synaptopathy Without Permanent Threshold Shift

While CIND is questionable as the major problem resulting from NIS, temporal processing deficits have been demonstrated as being associated with NIS in both animal and human subjects. Auditory system signal processing is highly distinguishable from that of other sensory systems, such as vision, due to its high temporal resolution (Hirsh, 1959; Ronken, 1970; Leshowitz, 1971). Temporal processing disorders have been reported in subjects with presbycusis (Schneider and Hamstra, 1999; Pichora-Fuller and Souza, 2003; Gordon-Salant, 2005; Martin and Jerger, 2005; Walton, 2010; Humes et al., 2012), also known as age-related hearing loss, and in subjects with auditory neuropathy (Kumar and Jayaram, 2005; Vlastarakos et al., 2008; Narne, 2013; Lobarinas et al., 2020). Since NIS is a type of auditory neuropathy, temporal processing difficulties are likely to occur in subjects with NIS.

The pathological locus of NIS is the ribbon synapses between the IHCs and the SGNs, which happens to be the first speed-limiting site of auditory processing along the ascending auditory pathway. It is well recognized that the primary function of the presynaptic ribbons is to facilitate neurotransmission through the synapses (Moser et al., 2006; Safieddine et al., 2012; Moser and Starr, 2016). Therefore, damage to ribbons would be expected to give rise to limitations of auditory processing speed (Buran et al., 2010; Jing et al., 2013), likely resulting in temporal processing disorders.

In a single unit study, a development of temporal processing deficits was clearly associated with ribbon synapse repair after a damaging noise exposure (Song et al., 2016). In this study, a noise exposure of 105 dB SPL for 2 h was given to albino Guinea pigs. This noise led to an initial synaptic loss of approximately 50% in the high frequency region. Within the month following the noise exposure, temporal coding deficits developed along with partial recovery of the number of ribbon synapses. The temporal coding deficits manifested as a delayed onset peak of ANF firing as well as a reduced peak rate. Since the deficits were not seen shortly after the noise exposure, but only a month later after the synaptic count had largely recovered, it was concluded that the repaired synapses had presented problems with encoding signal onset (Song et al., 2016). A second study executed by the same researchers reported similar temporal processing deficits as measured in ABR and CAP in guinea pigs exposed to the same noise (Shi et al., 2013).

Temporal processing disorders resulting from noise exposure have also been investigated in human participants, both objectively and behaviorally. For instance, past objective studies have used ABR wave V in order to identify temporal coding deficits in humans following noise exposure (Mehraei et al., 2016; Prendergast et al., 2017a). In the study by Mehraei et al. (2016), it was found that the masking-induced wave-V latency shifts were correlated with changes in ABR wave-I amplitude, which may reflect the number of functional ANFs. In the mice observed in the study, it was demonstrated that NIS reduced wave-I amplitude growth with sound level. Notably, the amount of wave-V latency shift in noise was also reduced. Among the human participants in this study, those with small masking-induced wave-V latency shifts (which likely would be associated with smaller ABR wave-I amplitude and a larger loss of synapses) performed poorer on a sound localization task requiring discrimination of interaural time differences (ITD) in sound envelopes (Mehraei et al., 2016). This result suggests that NIS may result in temporal processing deficits. In another objective study, a correlation was found between poor envelope following responses (EFR) and poor ITD threshold, which was representative of poor temporal resolution (Bharadwaj et al., 2015). Poor EFR (i.e., reduced amplitude and/or phase-locking value) has also been reported as evidence of temporal processing disability in subjects with NIS (Bharadwaj et al., 2014; Shaheen et al., 2015; Parthasarathy and Kujawa, 2018; Prendergast et al., 2019).

A connection between CIND and temporal processing disorders has also been found in humans from behavioral studies. In one study, Snell et al. found that individuals with poorer gap detection thresholds showed significantly poorer word scores as the level of background babble increased (Snell et al., 2002), suggesting that temporal processing could play an important role in understanding speech in noise. More evidence is available for temporal processing deficits with NIS. In one study, participants who had been exposed to noise had trouble discriminating a temporally fluctuating noise from a more stationary noise than those without noise exposure (Stone et al., 2008). In another study, noise-exposed train drivers were found to perform poorer than controls in various tests of temporal processing ability, including gap detection, modulation detection and duration pattern detection. The poorer temporal resolution was also correlated with poor speech recognition in noise (Kumar et al., 2012). In light of evidence for the functional role of ribbon synapses in temporal processing and the sensitivity of the synapse to noise, as well as the apparent connection between temporal processing deficits and difficulty of hearing in noise, it is reasonable to assume that noise damage may cause CIND by degrading temporal processing.

However, reports refuting the connection between temporal processing deficits and CIND from NIS also exist. For instance, one study examined the auditory processing abilities of middle-aged participants with normal hearing thresholds by measuring AM detection thresholds. In this study, no clear relationship between noise exposure and auditory perception was found (Yeend et al., 2017). In another study, a significant but weak correlation was found between speech-in-noise deficits and temporal processing deficits in noise-exposed groups with normal hearing thresholds (Prendergast et al., 2017b). In both of those reports with negative results, the subjects in the noise group were selected based upon self-report and might not have had NIS.

### Is Temporal Processing Disorder a Concern for Evaluating Coding-in-Noise Deficit?

The review above suggests that temporal processing deficits are likely to occur in individuals with NIS and may give rise to speech-in-noise deficits (or CIND). Logically, the evaluation of CIND should take temporal processing deficits into account, since temporal processing is involved in the detection of signals in background noise. As outlined in section “Noise Induced Synaptopathy Studies in Animal Models and Difficulty in Translation,” the real-world noise experienced by humans tends to be temporally modulated. In such noise, it is possible to detect signals in the dips of the masker. However, such listening in the dips likely depends on robust temporal processing. To mimic real life hearing in noise challenges, maskers used in experiments investigating coding ability in background noise should also be temporally modulated. However, this issue has received scant attention in research designs—particularly in studies of CIND with NIS in animal models.

In behavioral studies with human participants, both stationary and modulated maskers (such as multi-talker babble) have been used in speech-in-noise tests in order to examine potential deficits in subjects with NIS, but the temporal characteristics of the masker have received insufficient focus, and there are no comprehensive comparisons of the masking effect from maskers with varying temporal features. For example, in one study reviewed above, stationary noise was used as the masker and no differences were found between the noise-exposed group and the control group in the speech-in-noise task (Prendergast et al., 2017b). In another study examining the effect of noise-induced tinnitus on speech-in-noise understanding in young adults, participants with noise-induced tinnitus showed worse speech-in-noise performance than non-tinnitus controls regardless of whether the masker was stationary or modulated (Gilles et al., 2016). However, there was no control group without noise exposure used in this study. Only the study by Kumar et al. (2012) appeared to confirm worse speech-in-noise performance in noise-exposed participants by using multi-talker babble as the masker (Kumar et al., 2012). However, the masking effect of the multi-talker babble was not compared to a stationary masker. It is therefore evident that a valid comparison cannot be made across the available studies to differentiate the effects of masker types (stationary versus modulated). To date, there are no comprehensive evaluations of whether a temporally modulated masker is superior to a stationary masker in a speech-in-noise test used to identify CIND in subjects with NIHHL.

## Is the Synaptic Damage and Repair Responsible for the Temporary Threshold Shift and Recovery?

Cochlear threshold recovery after a non-PTS-inducing noise exposure co-occurs with synapse count recovery and/or repair of damaged synapses. This co-occurrence has been considered by some researchers as evidence supporting synaptic repair as the *mechanism* of temporary threshold shift (TTS) recovery (Robertson, 1983; Puel et al., 1997; Shi et al., 2015; Wang et al., 2015). However, this idea conflicts with our understanding of the physiological mechanisms that determine cochlear threshold. It is well recognized that noise-induced reductions in auditory sensitivity are mainly due to damage to outer hair cells (OHC), which provide active gain for soft sounds (Hudspeth, 1997; Szalai et al., 2011). Threshold recovery following TTS is associated with a full recovery of OHC function, demonstrated by a recovery of otoacoustic emissions (OAE) (Subramaniam et al., 1994; Chang and Norton, 1996; Kujawa and Liberman, 2009) and cochlear microphonics (CM) (Wang et al., 1992, 2011; Chen et al., 1995; Chen and Liu, 2005; Chen and Zhao, 2007). In addition, repair of stereocilia and the tectorial membrane have been considered as potential mechanisms underlying the resolution of TTS in several studies (Sohmer, 1997; Nordmann et al., 2000; Wang et al., 2002, 2011; Tsuprun et al., 2003). To the extent that noise-induced damage to OHCs and surrounding structures is reversible, this reversibility provides a reasonable account for the recovery of cochlear thresholds following noise exposure.

Noise-induced IHC and synapse damage and repair are less likely to be involved in threshold recovery. Each IHC is innervated by more than 10 SGNs, and noise damage tends to be selective to synapses innervating high-threshold fibers that have low spontaneous spike rates (SR) (Furman et al., 2013; Song et al., 2016). Damage/repair or disruption of these synapses should not result in any change in thresholds, similar to results obtained *via* ouabain-induced cochlear damage at low doses (Bourien et al., 2014). This is further supported by differences in the time courses for the recovery of ABR threshold and CAP amplitude, which are related to the total number of ANFs that are functional. In a series of experiments using Guinea pigs, we found that ABR threshold shifts induced by brief noise exposures at 106 dB SPL were completely recovered a week later, with continuing recovery of CAP amplitudes and synapse counts occurring well after that time point. The hypothesis that moderate damage to IHCs and their synapses with SGNs may not impact thresholds is also supported by the finding that up to a 60% loss of SGNs, due to the selective IHC death induced by carboplatin in chinchillas, does not affect cochlear thresholds (Salvi et al., 2016).

Extant data cannot fully rule out changes in synaptic sensitivity that may occur in parallel with damage and repair of OHCs and surrounding structures. Since OHCs provide positive feedback in sound conduction, such changes in synaptic sensitivity would need to be observed by stimulation bypassing these OHC-based effects (to rule out the slim possibility that a temporary reduction in synaptic sensitivity is responsible for the noise-induced TTS).

## Temporary Conclusion and Future Tasks

**FIGURE 3:**
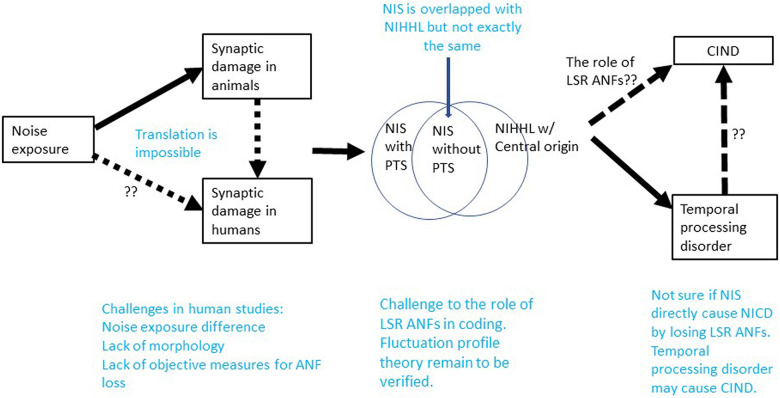
Summary on what we know and what we do not know in the research of NIS and NIHHL. Note that NIS without PTS is overlapped with NIHHL of peripheral origin. The dashed lines without question markers indicate where the connection remain to be speculation and need to be verified.

Figure 3 presents a summary of this review in the attempt to show what we current know as well as gaps in our knowledge concerning NIS and NIHHL.

Conceptually, NIS is largely but incompletely overlapped with NIHHL, since NIS can occur with or without PTS. While NIS refers to the peripheral effect of noise, NIHHL should also cover problems with central origin.

Studies of NIS and NIHHL have received wide interest for more than 10 years since the discovery of substantial ribbon synapse loss following a brief noise exposure that did not cause PTS in CBA mice (Kujawa and Liberman, 2009). Unlike what was seen in this earliest report, many studies have found that the initial synapse loss can be partially recovered. Based upon two available single unit studies in Guinea pigs, the noise damage appears to be biased to synapses innervating LSR ANFs (Furman et al., 2013; Song et al., 2016). However, in one of the two reports, this issue was observed dynamically, and the result showed that a normal distribution of ANFs across SR category was re-established with the partial recovery of total synaptic count.

Translation of animal data on NIS to humans is challenging due to the large differences in noise exposure used in animal research studies and the noise experienced in real human life. Hypothesized NIS in humans is also difficult to confirm due to a lack of morphological data and reliable objective tests that can quantify a loss of ANF function.

Functionally, CIND has been considered to be the major functional problem associated with NIS and NIHHL based upon the theory of SR-based ANF categorization and the finding of a disproportionate loss of LSR ANF after noise exposure. However, CIND has yet to be confirmed as a consequence of NIS in either animals or humans, suggesting a possible problem with the hypothesis. The hypothesis that speech encoding (and speech-in-noise encoding) is compromised in NIS because it depends disproportionately on LSR ANFs, which are selectively damaged by noise exposure, is further challenged by the fluctuation profile model. This model contends that speech is more robustly encoded by fluctuation profiles conveyed *via* HSR ANFs, and that LSR ANFs play a more important role in efferent control *via* the LOC and MOC. However, there are several unresolved issues for this model that remain to be validated, including the role of MOC function in this model.

Temporal processing disorders have been shown to be the most likely functional deficit associated with NIS, and these may be connected to hearing difficulties in noise, particularly with temporally modulated maskers. However, further research is required in humans, with particular attention paid to: (1) better quantifications of noise exposure and consistent use of control groups, (2) better quantifications or evaluations of NIS, (3) careful comparisons of maskers with different temporal characteristics to allow for the evaluation of the impact of temporal processing deficits on hearing in noise.

## Author Contributions

SR, ZZ, and SA: conceptualization, visualization, and writing. LX and JW: conceptualization, visualization, writing, and funding acquisition. All authors: contributed to the article and approved the submitted version.

## Conflict of Interest

The authors declare that the research was conducted in the absence of any commercial or financial relationships that could be construed as a potential conflict of interest.

## Publisher’s Note

All claims expressed in this article are solely those of the authors and do not necessarily represent those of their affiliated organizations, or those of the publisher, the editors and the reviewers. Any product that may be evaluated in this article, or claim that may be made by its manufacturer, is not guaranteed or endorsed by the publisher.

## Abbreviations

ABR: auditory brainstem response
AMD: amplitude modulation detection
ANFs: auditory nerve fibers
ANCOVAs: analyses of covariance
CAP: compound action potential
CF: characteristics frequencies
CIND: coding-in-noise deficit
CN: cochlear nucleus
AVCN: anterior ventral CN
PCVN: posterior ventral CN
DCN: dorsal CN
CRM: co-ordinate response measure
DPOAE: distortion-product otoacoustic emission
DTT: digit triplet test
EAR: efferent acoustic reflex
ECochG: electrocochleography
EFR: envelope following response
HC: hair cells
IHC: inner HC
OHC: outer HC
HIN: hearing-in-noise
IPD: interaural phase difference
ITD: interaural time differences
MEMR: middle ear muscle reflex
NESI: Noise Exposure Structured Interview
NIS: noise induced synaptopathy
NIHHL: noise induced hidden hearing loss
OC: olivocochlear
MOC: medial OC
LOC: lateral OC
PSTH: peristimulus time histograms
PTA: pure tone average
PTS: permanent threshold shift
SR: spontaneous rate
L/M/HSR: low/medial/high SR
SGN: spiral ganglion neuron
SPiN: speech perception in noise
(T)MTF: (temporal) modulation transfer function
TTS: temporary threshold shift.
